# Supplementary material for: Effector loss drives adaptation of Pseudomonas syringae pv. actinidiae biovar 3 to Actinidia arguta
Source: PLoS Pathog. 2022 May 27;18(5):e1010542. doi: 10.1371/journal.ppat.1010542 (PMC9182610; doi:10.1371/journal.ppat.1010542)
Supplement: S1 Table — (DOCX) [file ppat.1010542.s001.docx]

**Table S1 | Plasmid cloning and confirmation primers used in this study.**

| Gene | | Sense | Primer Sequence (5’-3’) | Product size | | Reference |
| --- | --- | --- | --- | --- | --- | --- |
| *Psa-hopA1* | | A1F2 | GCCTCGATGTCGGCGC | 132 bp | | [1] |
|  |  | A1R1 | ATTCGATAGAAGAACTTCTTTGCGTTT |  |  |  |
| *Psa-hopZ5* | | Z5F2 | ACAACTTCAGGCTACAATACTTACGC | 102 bp | | [1] |
|  |  | Z5R2 | CTCAGGATGCGTTTCGGTTAC |  |  |  |
| *Psa-X27* | | Forward | CAGGGCTGCCGATTTGATTC | 1804 bp | | This study |
|  | | Reverse | CAAAGGCTATGACGCAGAACAC |  |  |  |
| *Psa-ITS* | | PsaF1 | TTTTGCTTTGCACACCCGATTTT | 280 bp | | [2] |
|  |  | PsaR2 | CACGCACCCTTCAATCAGGATG |  |  |  |
| *Psa-ompP1* | | Forward | CACGATACATGGGCTTATGC | 492 bp | | [3] |
|  |  | Reverse | CTTTTCATCCACACACTCCG |  |  |  |
| *AcEF1α* | | Forward | GCACTGTCATTGATGCTCCT | 118 bp | | [4] |
|  |  | Reverse | CCAGCTTCAAAACCACCAGT |  |  |  |
| *hopAF1b_check* | | Forward | ATGGTTTCTTTAGACGCTTATC | 642 bp | | This study |
|  |  | Reverse | TTAGCCAGTCACCAAATGTT |  |  |  |
| *hopD2a_check* | | Forward | ATGCAGAATCATGTCATTACTT | 1020 bp | | This study |
|  |  | Reverse | CTAAAAGCGTTGTTGAGAGG |  |  |  |
| *hopAW1a_check* | | Forward | ATGCGCGTGAGAGTATCAAAC | 663 bp | | This study |
|  | | Reverse | TTACGAGCGCACAGGCAGAA |  | |  |
| *hopF1e_check* | Forward | | GTGGGCAATATTTGTGGTAC | 843 bp | This study | |
|  | Reverse | | CTATCCATCAGACCGAGAAT |  |  | |
| *hopAW1a* | | Forward | CCCTCTGGGTAAAACTGGCG | 885 bp | | This study |
|  | | Reverse | CCCGAGCTGATGCGCAT |  | |  |
| *hopF1c* | Forward | | GCCTGTCCTCAACGAAAGATCATC | 1225 bp | This study | |
|  | Reverse | | CACCGAAAAACCCTCAACATGC |  |  |  |
| *hopZ5a* | Forward | | GACAAGCCAGCGATAACACCTA | 1455 bp | This study | |
|  | Reverse | | CGCTCACGAGAAAGTCTCAATTG |  |  |  |
| *avrRpm1a* | Forward | | GCGTGTCCAGACCTGTAGATTT | 876 bp | This study | |
|  | Reverse | | TCGGTTTTTTCAGACGAATTCTTGAA |  |  |  |

**References**

1**.** Andersen MT, Templeton MD, Rees-George J, Vanneste JL, Cornish DA, Yu J, et al. Highly specific assays to detect isolates of *Pseudomonas syringae* pv. *actinidiae* biovar 3 and *Pseudomonas syringae* pv. *actinidifoliorum* directly from plant material. Plant Pathol. 2017;67:1220-1230.

2. Rees-George J, Vanneste JL, Cornish DA, Pushparajah IPS, Yu J, Templeton MD, et al. Detection of *Pseudomonas syringae* pv. *actinidiae* using polymerase chain reaction (PCR) primers based on the 16S-23S rDNA intertranscribed spacer region and comparison with PCR primers based on other gene regions. Plant Pathol. 2010;59(3):453-64.

3. Laflamme B, Middleton M, Lo T, Desveaux D, Guttman DS. Image-based quantification of plant immunity and disease. Mol Plant-Microbe Interact. 2016;29(12):919-24.

4. Nardozza S, Boldingh HL, Osorio S, Höhne M, Wohlers M, Gleave AP, et al. Metabolic analysis of kiwifruit (*Actinidia deliciosa*) berries from extreme genotypes reveals hallmarks for fruit starch metabolism. J Exp Bot. 2013;64(16):5049-63.
