# Supplementary material for: Effector loss drives adaptation of Pseudomonas syringae pv. actinidiae biovar 3 to Actinidia arguta
Source: PLoS Pathog. 2022 May 27;18(5):e1010542. doi: 10.1371/journal.ppat.1010542 (PMC9182610; doi:10.1371/journal.ppat.1010542)
Supplement: S2 Table — (DOCX) [file ppat.1010542.s002.docx]

**Table S2. Transgenic Psa3 V-13 effector knockout and plasmid-complemented strains.**

| **Strain** | **Description** | **Size of deletion (bp)** | **Source** |
| --- | --- | --- | --- |
| Psa3 V-13 Δ*hrcC* | deleted *hrcC* | - | [1] |
| Psa3 V-13 Δ*CEL* | deleted *hopN1a, shcM*, *hopM1f, hrpW1, shcE* and *avrE1d* | 14,168 | [2] |
| Psa3 V-13 Δ*hopR1* | deleted *hopR1b* (extended region owing to flanking repeat sequences) | 7,906 | [2] |
| Psa3 V-13 Δ*sEEL* | deleted *hopAW1a, hopF1e* (and associated *shcF*)*, hopD2a, hopAF1b,* and *hopF1a* (and associated *shcF*) | 11,479 | This study |
| Psa3 V-13 Δ*fEEL* | deleted *avrD, hopF4a* (and associated *shcF*)*, avrB2b, hopAW1a, hopF1e* (and associated *shcF*)*, hopD2a, hopAF1b, and hopF1e* (and associated *shcF*) | 28,594 | This study |
| Psa3 V-13 Δ*xEEL* | *deleted hopQ1a, hopD1a, avrD, hopF4a* (and associated *shcF*)*, avrB2b, hopAW1a, hopF1e* (and associated *shcF*)*, hopD2a, hopAF1b,* and *hopF1a* (and associated *shcF*) | 38,844 | This study |
| Psa3 V-13 Δ*tEEL* | deleted *hopF1e* (and associated *shcF*)*, hopD2a, hopAF1b,* and *hopF1a* (and associated *shcF*) | 9,845 | This study |
| Psa3 V-13 Δ*hopAW1a* | deleted *hopAW1a* | 707 | This study |
| Psa3 V-13 Δ*hopZ5a/*Δ*hopH1a* | deleted *hopZ5a* and *hopH1a* | 2,128 | This study |
| Psa3 V-13 Δ*hopAM1-1*/Δ*hopAM1-2* | deleted *hopAM1a-1* (extended region) and *hopAM1a-2*; (two separate loci) | 6,913 + 2,640 | This study |
| Psa3 V-13 Δ*hopQ1* | deleted *hopQ1a* | 1,370 | This study |
| Psa3 V-13 Δ*hopD1* | deleted *hopD1a* | 2,201 | This study |
| Psa3 V-13 Δ*hopI1* | deleted *hopI1c* | 1,096 | This study |
| Psa3 V-13 Δ*hopY1* | deleted *hopY1b* | 878 | This study |
| Psa3 V-13 Δ*avrRpm1a* | deleted *avrRpm1a* | 738 | This study |
| Psa3 V-13 Δ*hopW1c* | deleted *hopW1c* (extended region owing to flanking repeat sequences) | 5,979 | This study |
| Psa3 V-13 Δ*hopBN1a* | deleted *hopBN1a* (and associated *shcF*) | 1,436 | This study |
| Psa3 V-13 Δ*hopAZ1a* | deleted *hopAZ1a* | 681 | This study |
| Psa3 V-13 Δ*hopF1c* | deleted *hopF1c* (and associated *shcF*; extended region owing to flanking repeat sequences) | 6,655 | This study |
| Psa3 V-13 Δ*hopAU1a* | deleted *hopAU1a* | 2,331 | This study |
| Psa3 V-13 Δ*hopBP1a* | deleted *hopBP1a* | 1,270 | This study |
| Psa3 V-13 Δ*hopAS1b* | deleted *hopAS1b* | 4,109 | This study |
| Psa3 V-13 Δ*avrPto1b* | deleted *avrPto1b* | 526 | This study |
| Psa3 V-13 Δ*hopS2b* | deleted *hopS2b* (and associated *shcS2*) | 1,237 | This study |
| Psa3 V-13 Δ*hopZ5a* | deleted *hopZ5a* | 1,016 | This study |
| Psa3 V-13 Δ*sEEL* + pBBR1MCS-5B:avrRps4_pro_:*hopF1e*:HA | Plasmid-complemented with *hopF1e* | - | This study |
| Psa3 V-13 Δ*sEEL* + pBBR1MCS-5B:avrRps4_pro_:*hopD2a*:HA | Plasmid-complemented with *hopD2a* | - | This study |
| Psa3 V-13 Δ*sEEL* + pBBR1MCS-5B:avrRps4_pro_:*hopAF1b*:HA | Plasmid-complemented with *hopAF1b* | - | This study |
| Psa3 V-13 Δ*hopAW1a* + pBBR1MCS-5B:avrRps4_pro_:*hopAW1a*:HA | Plasmid-complemented with *hopAW1a* | - | This study |
| Psa3 V-13 Δ*hopZ5a* + pBBR1MCS-5B:avrRps4_pro_:*hopZ5a*:HA | Plasmid-complemented with *hopZ5a* | - | This study |
| Psa3 V-13 Δ*avrRpm1a* + pBBR1MCS-5B:avrRps4_pro_:*avrRpm1a*:HA | Plasmid-complemented with *avrRpm1a* | - | This study |
| Psa3 V-13 Δ*hopF1c* + pBBR1MCS-5B:avrRps4_pro_:*shcF*:*hopF1c*:HA | Plasmid-complemented with *hopF1c* (with its naturally truncated chaperone *shcF*) | - | This study |
| Psa3 V-13 Δ*hopAW1a* + pBBR1MCS-5 | Plasmid-complemented with empty vector (EV) | - | This study |
| Psa3 V-13 Δ*hopZ5a* + pBBR1MCS-5 | Plasmid-complemented with empty vector (EV) | - | This study |
| Psa3 V-13 Δ*avrRpm1a* + pBBR1MCS-5 | Plasmid-complemented with empty vector (EV) | - | This study |
| Psa3 V-13 Δ*hopF1c* + pBBR1MCS-5 | Plasmid-complemented with empty vector (EV) | - | This study |

**References**

1. Straub C, Colombi E, Li L, Huang H, Templeton MD, McCann HC, et al. The ecological genetics of *Pseudomonas syringae* from kiwifruit leaves. Environ Microbiol. 2018;20(6):2066-84.

2. Jayaraman J, Yoon M, Applegate ER, Stroud EA, Templeton MD. AvrE1 and HopR1 from *Pseudomonas syringae* pv. *actinidiae* are additively required for full virulence on kiwifruit. Mol Plant Pathol. 2020;21(11):1467-80.
