# Supplementary material for: Effector loss drives adaptation of Pseudomonas syringae pv. actinidiae biovar 3 to Actinidia arguta
Source: PLoS Pathog. 2022 May 27;18(5):e1010542. doi: 10.1371/journal.ppat.1010542 (PMC9182610; doi:10.1371/journal.ppat.1010542)
Supplement: S3 Table — All wild-type Psa strains were sourced from the International Collections of Micro-organisms from Plants (ICMP) or the National Agriculture and Food Research Organization (NARO); (designated MAFF). (DOCX) [file ppat.1010542.s003.docx]

**Table S3. Wild-type Psa strains.** All wild-type Psa strains were sourced from ICMP/MAFF.

| Biovar | Strain | Collection number | GenBank accession number | Country of origin | Year | Host plant | Reference |
| --- | --- | --- | --- | --- | --- | --- | --- |
| Psa1 | **J-35** | [ICMP 9617](https://scd.landcareresearch.co.nz/Specimen/ICMP%209617?collection=ICMP&searchCollection=ICMP&query=9617&currentDisplayTab=list&pageNumber=0&sortField=relevance) | [CM002753](https://www.ncbi.nlm.nih.gov/nuccore/CM002753.1/) | Japan | 1984 | *Actinidia chinensis* var. *deliciosa* ‘Hayward’ | [1] |
| Psa2 | **K-28** | [ICMP 19071](https://scd.landcareresearch.co.nz/Specimen/ICMP%2019071?collection=ICMP&searchCollection=ICMP&query=19071&currentDisplayTab=list&pageNumber=0&sortField=relevance) | [NZ_RBSG00000000](https://www.ncbi.nlm.nih.gov/nuccore/NZ_RBSG00000000) | Korea | 1997 | *Actinidia chinensis* | [1] |
| Psa3 | **V-13** | [ICMP 18884](https://scd.landcareresearch.co.nz/Specimen/ICMP%2018884?collection=ICMP&searchCollection=ICMP&query=18884&currentDisplayTab=list&pageNumber=0&sortField=relevance) | [CP011972-3](https://www.ncbi.nlm.nih.gov/nuccore/CP011972) | New Zealand | 2010 | *Actinidia chinensis* var. *deliciosa* ‘Hayward’ | [2] |
| Psa3 | **X-27** | [ICMP 24332](https://scd.landcareresearch.co.nz/Specimen/ICMP%2024332?collection=ICMP&searchCollection=ICMP&query=24332&currentDisplayTab=list&pageNumber=0&sortField=relevance) | [SRR16648333](https://trace.ncbi.nlm.nih.gov/Traces/sra?run=SRR16648333) | New Zealand | 2017 | *Actinidia arguta* ‘HortGem Tahi’ | This study. |
| Psa3 | **10627** | N/A | - | New Zealand | 2010 | *Actinidia chinensis* | [3] |
| Psa5 | **-** | [MAFF 212057](https://www.gene.affrc.go.jp/databases-micro_search_detail_en.php?maff=212057&width=500&height=200) | [JAAEYO010000000](https://www.ncbi.nlm.nih.gov/nuccore/JAAEYO000000000.1/) | Japan | 2012 | *Actinidia chinensis* var. *chinensis* ‘Hort16A’ | [4] |
| Psa6 | **-** | [MAFF 212134](https://www.gene.affrc.go.jp/databases-micro_search_detail.php?maff=212134&width=500&height=200) | [MSBW01000000](https://www.ncbi.nlm.nih.gov/nuccore/MSBW00000000.1) | Japan | 2015 | *Actinidia chinensis* var. *deliciosa* | [5,6] |

References

1. McCann HC, Rikkerink EHA, Bertels F, Fiers M, Lu A, Rees-George J, et al. Genomic analysis of the kiwifruit pathogen *Pseudomonas syringae* pv. *actinidiae* provides insight into the origins of an emergent plant disease. PLoS Path. 2013;9(7):e1003503.
2. Templeton MD, Warren BA, Andersen MT, Rikkerink EH, Fineran PC. Complete DNA sequence of *Pseudomonas syringae* pv. *actinidiae*, the causal agent of kiwifruit canker disease. Genome announcements. 2015;3(5):e01054-15.
3. Vanneste JL, Yu J, Cornish DA, Tanner DJ, Windner R, Chapman JR, et al. Identification, virulence, and distribution of two biovars of *Pseudomonas syringae* pv. *actinidiae* in New Zealand. Plant Dis. 2013;97(6):708-19.
4. Fujikawa T, Sawada H. Genome analysis of the kiwifruit canker pathogen *Pseudomonas syringae* pv. *actinidiae* biovar 5. Scientific Reports. 2016;6:e21399.
5. Fujikawa T, Sawada H. Genome analysis of *Pseudomonas syringae* pv. *actinidiae* biovar 6, which produces the phytotoxins, phaseolotoxin and coronatine. Scientific reports. 2019;9(1):1-11.
6. Sawada H, Kondo K, Nakaune R. Novel biovar (biovar 6) of *Pseudomonas syringae* pv. *actinidiae* causing bacterial canker of kiwifruit (*Actinidia deliciosa*) in Japan. Japanese Journal of Phytopathology. 2016;82(2):101-115.
