# Supplementary material for: Effector loss drives adaptation of Pseudomonas syringae pv. actinidiae biovar 3 to Actinidia arguta
Source: PLoS Pathog. 2022 May 27;18(5):e1010542. doi: 10.1371/journal.ppat.1010542 (PMC9182610; doi:10.1371/journal.ppat.1010542)
Supplement: S4 Table — (DOCX) [file ppat.1010542.s004.docx]

**Table S4 | *Pseudomonas fluorescens* plasmid-complemented strains used in this study.**

| **Strain** | ***Description*** | **Source** |
| --- | --- | --- |
| *Pfo* (T3S) | *P. fluorescens* Pf0-1 carrying an artificial type III secretion system from *P. syringae* pv. *syringae* 61 | [1] |
| *Pfo* (WT) | *P. fluorescens* Pf0-1 WT strain | [1] |
| *Pfo* (T3S) or *Pfo* (WT) + EV | Plasmid-complemented with empty vector (pBBR1MCS-5) | [2] |
| *Pfo* (T3S) or *Pfo* (WT) + hopAW1a | Plasmid-complemented with *hopAW1a* (cloned under *avrRps4* promoter and tagged with HA) | This study |
| *Pfo* (T3S) or *Pfo* (WT) + hopZ5a | Plasmid-complemented with *hopZ5a* (cloned under *avrRps4* promoter and tagged with HA) | This study |
| *Pfo* (T3S) or *Pfo* (WT) + hopF1c | Plasmid-complemented with *hopF1c* (cloned under *avrRps4* promoter and tagged with HA) | This study |
| *Pfo* (T3S) or *Pfo* (WT) + avrRpm1a | Plasmid-complemented with *avrRpm1a* (cloned under *avrRps4* promoter and tagged with HA) | This study |
| *Pfo* (T3S) or *Pfo* (WT) + *hopA1j_Psy61_* | Plasmid-complemented with *shcA* and *hopA1j* from *P. syringae* pv. *syringae* 61 | [3] |

**References**

1. Thomas WJ, Thireault CA, Kimbrel JA, Chang JH. Recombineering and stable integration of the *Pseudomonas syringae pv. syringae* 61 hrp/hrc cluster into the genome of the soil bacterium *Pseudomonas fluorescens* Pf0‐1. The Plant Journal. 2009;60(5):919-28.

2. Jayaraman J, Yoon M, Applegate ER, Stroud EA, Templeton MD. AvrE1 and HopR1 from *Pseudomonas syringae* pv. *actinidiae* are additively required for full virulence on kiwifruit. Mol Plant Pathol. 2020;21(11):1467-80.

3. Jayaraman J, Chatterjee A, Hunter S, Chen R, Stroud EA, Saei H, et al. Rapid methodologies for assessing *Pseudomonas syringae* pv. *actinidiae* colonization and effector-mediated hypersensitive response in kiwifruit. Mol Plant-Microbe Interact. 2021;34(8): 880-890.
