# Supplementary figures and images for: Effector loss drives adaptation of Pseudomonas syringae pv. actinidiae biovar 3 to Actinidia arguta
Source: PLoS Pathog. 2022 May 27;18(5):e1010542. doi: 10.1371/journal.ppat.1010542 (PMC9182610; doi:10.1371/journal.ppat.1010542)

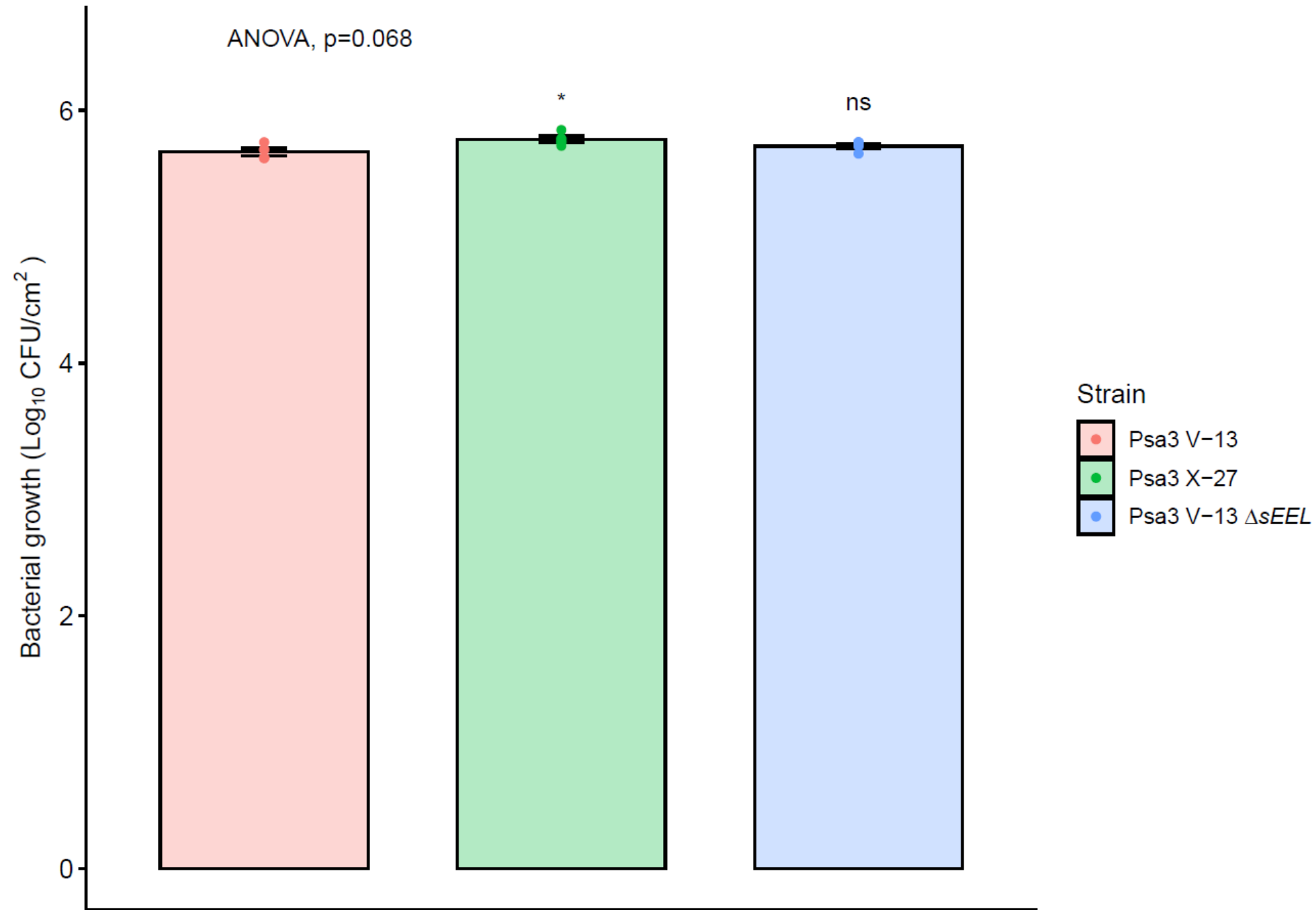

Supplement: S1 Fig — A. arguta AA07_03 plantlets were flood-inoculated with Psa3 V-13, Psa3 X-27, and Psa3 V-13 ΔsEEL at approximately 106 cfu/mL. Bar height represents the mean number of Log10 cfu/cm2 and error bars represent the standard error of the mean (SEM) between four pseudobiological replicates. (PDF) [file ppat.1010542.s005.pdf]

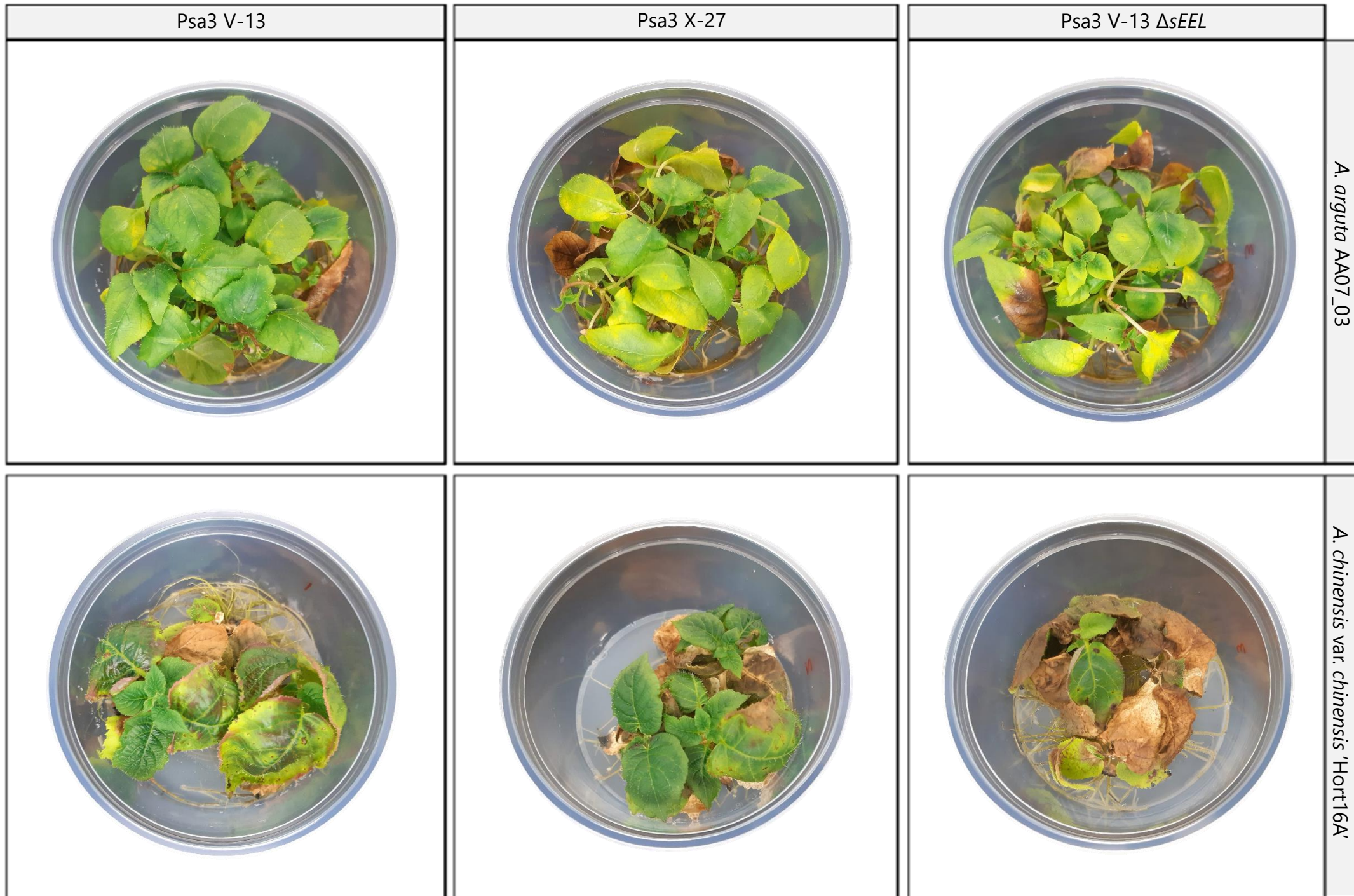

Supplement: S2 Fig — A. arguta AA07_03 kiwifruit plantlets were flood-inoculated at approximately 107 cfu/mL. Photographs of symptom development in representative pottles were taken at 50 days post-infection. (PDF) [file ppat.1010542.s006.pdf]

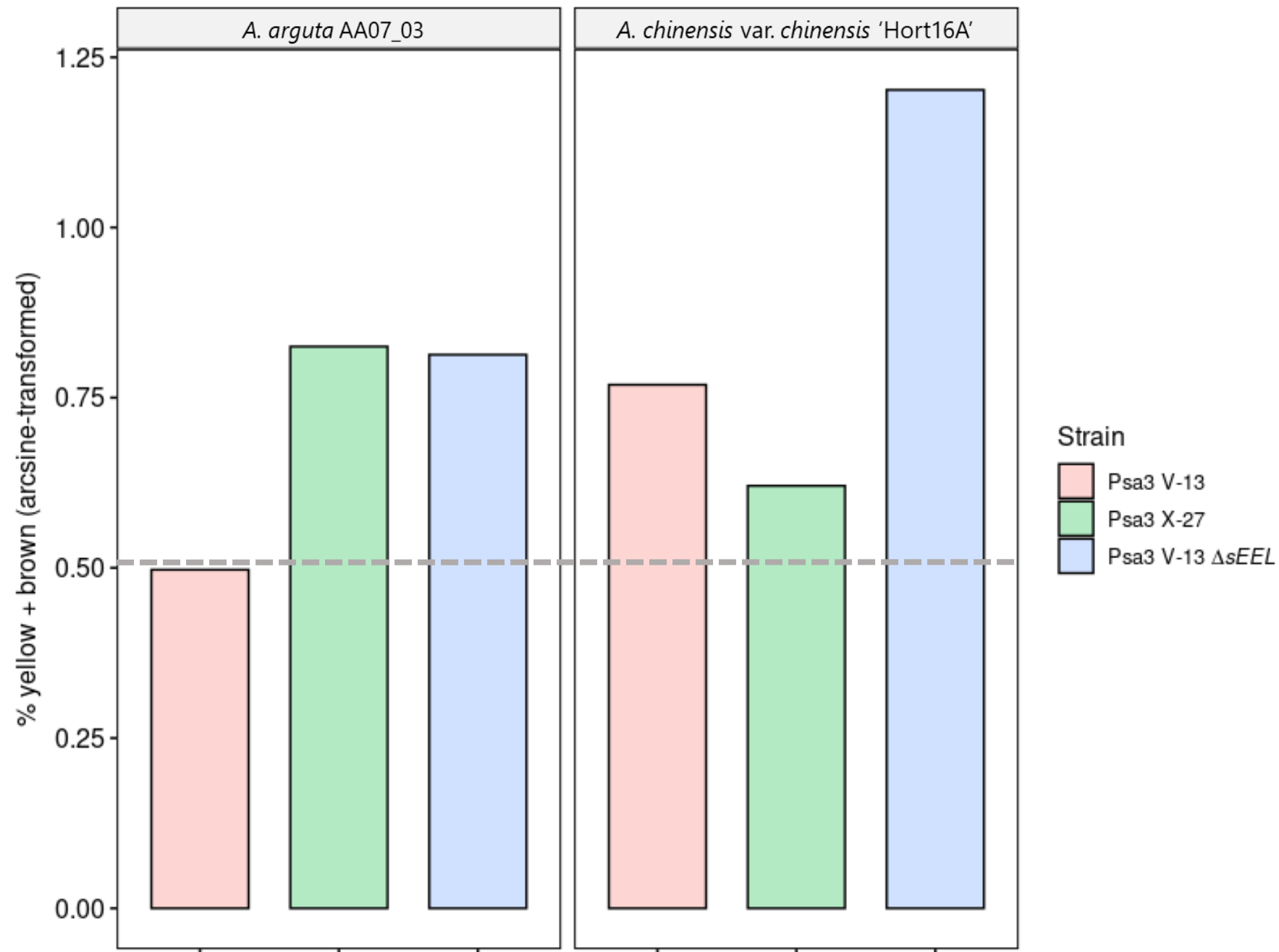

Supplement: S3 Fig — A modified PIDIQ image-based analysis of leaf yellowing and browning, expressed as a normalized arcsine-transformed percentage for symptomology photographs taken at 50 days post-infection (S2 Fig). Methodology adapted and modified from that in Laflamme, Dillon [70]. (PDF) [file ppat.1010542.s007.pdf]

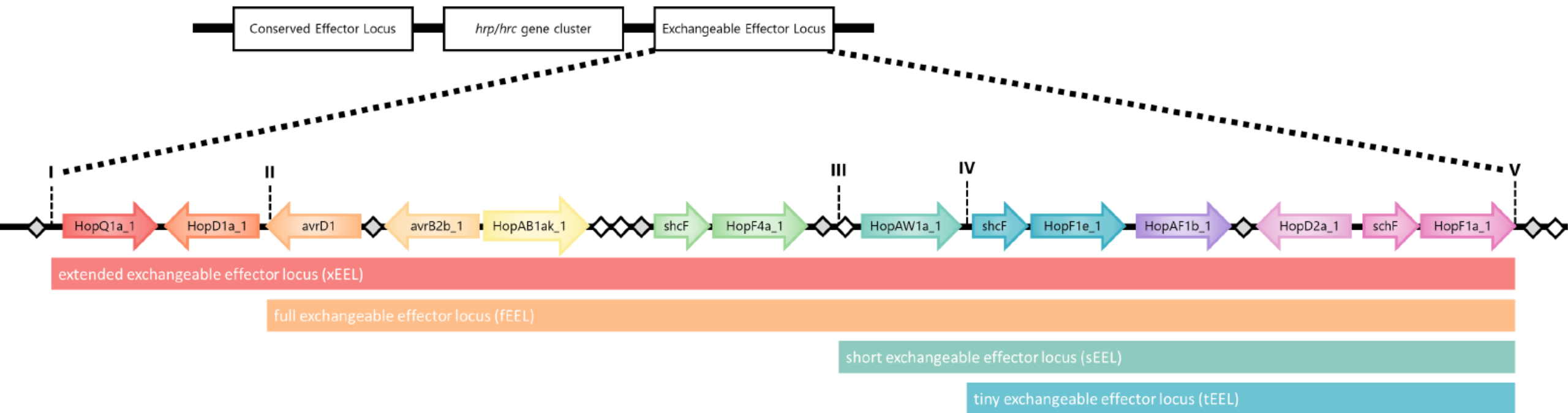

Supplement: S4 Fig — Schematic of the effectors comprising the xEEL (I-V; hopQ1a –hopF1a), fEEL (II-V; avrD1 –hopF1a), sEEL (III-V; hopAW1a –hopF1a), and tEEL (IV-V; hopF1e –hopF1a) loci in Psa3 V-13 ICMP 18884 strain are indicated. Potential recombination sites are indicated: Miniature Inverted Repeat Transposable Element (MITE; grey diamonds), DDE terminal inverted repeats (white diamonds). (PDF) [file ppat.1010542.s008.pdf]

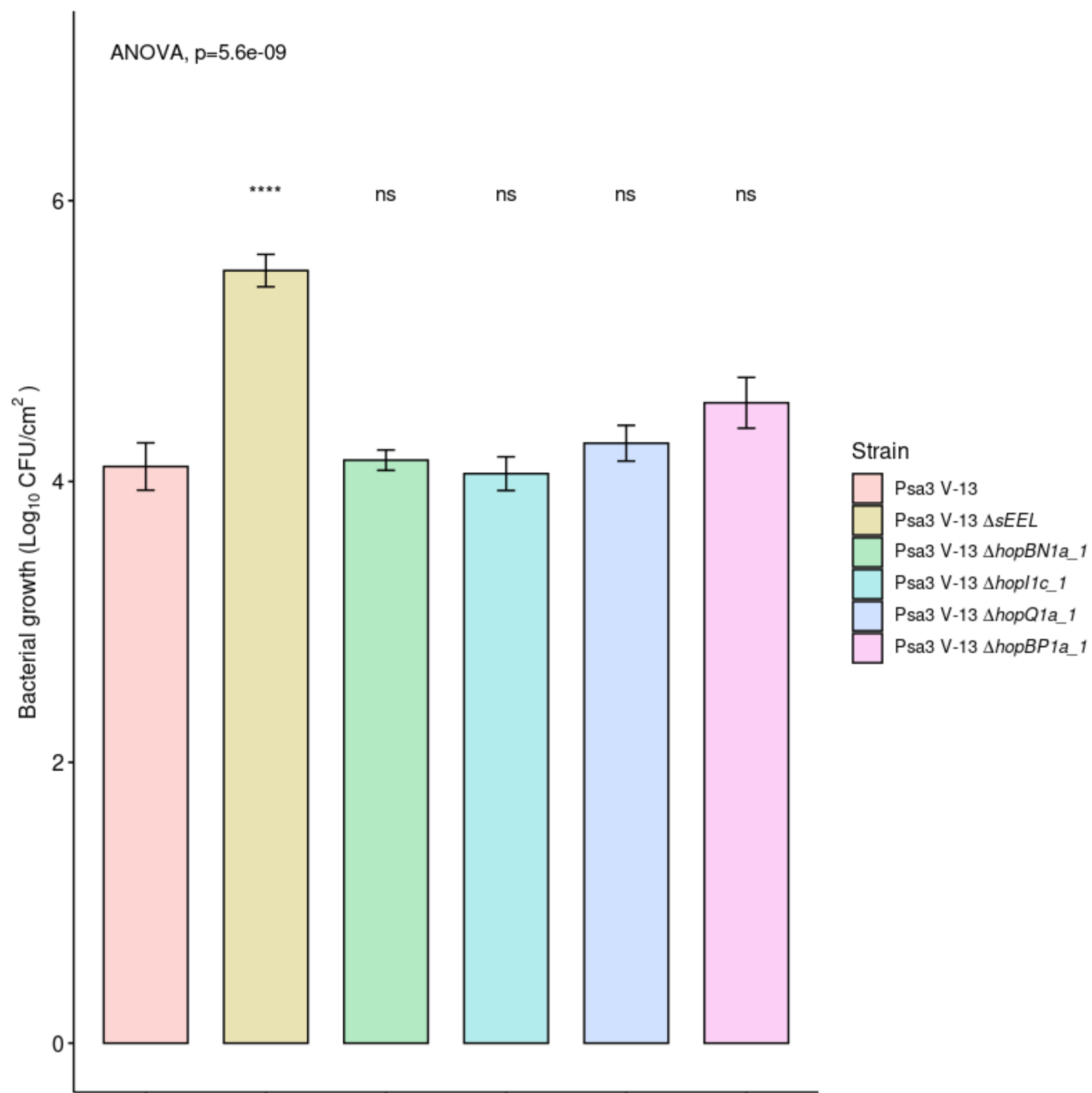

Supplement: S5 Fig — A. arguta AA07_03 kiwifruit plantlets were flood-inoculated at approximately 106 cfu/mL. Bacterial pathogenicity was quantified at 12 days post-inoculation relative to Psa3 V-13 using plate count quantification for four pseudobiological replicates, per strain, per experimental run and error bars represent the standard error of the mean (SEM). Asterisks indicate the statistically significant difference of Student’s t-test between the indicated strain and wild-type Psa3 V-13, where p≤.001 (****), and p>.05 (ns; not significant). This experiment was separately conducted twice (biological replicates) with two batches of independently grown plants and data were stacked to generate the bar graphs shown. (PDF) [file ppat.1010542.s009.pdf]

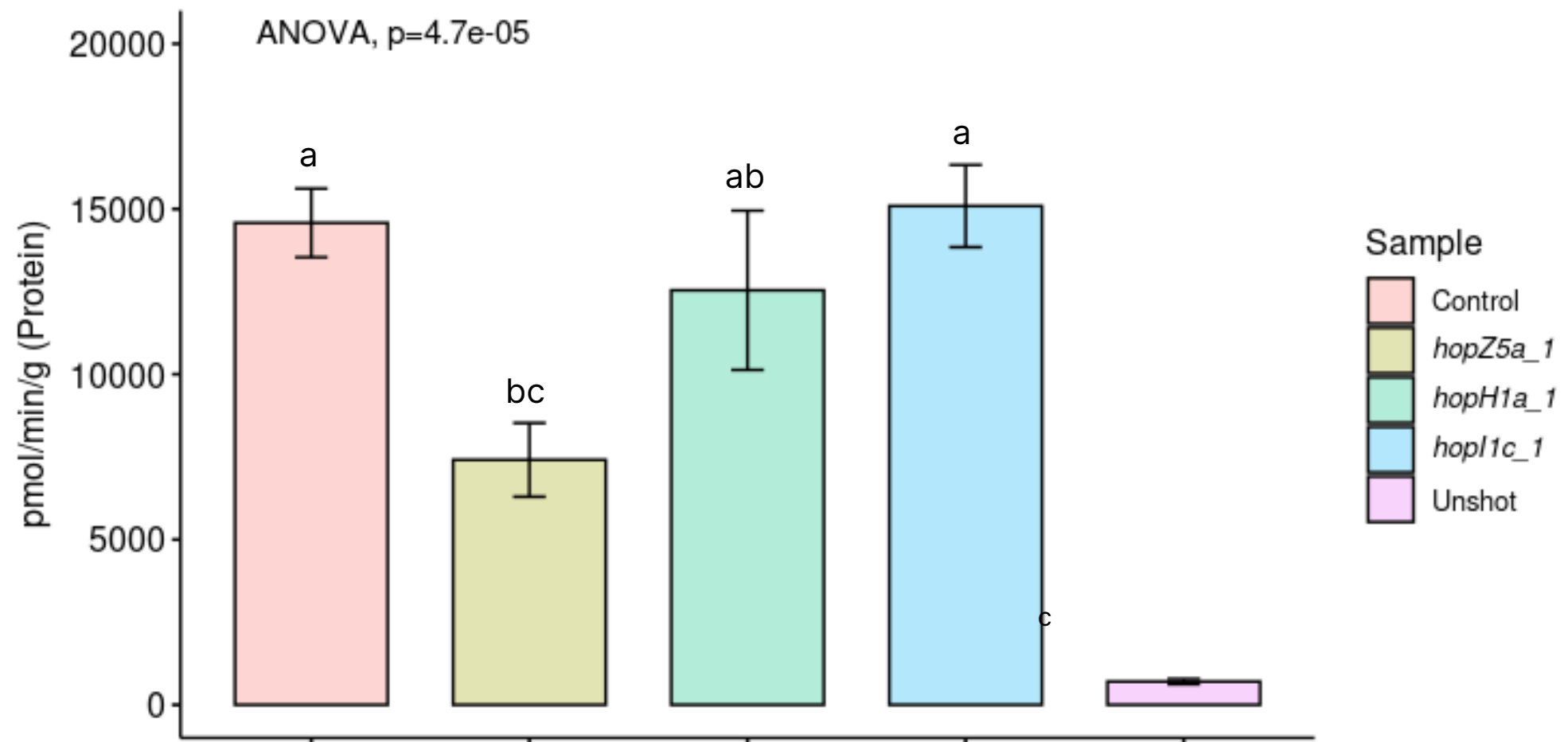

Supplement: S6 Fig — Avirulence effectors cloned into binary vector constructs tagged with GFP, or an empty vector (Control), were co-expressed with a β-glucuronidase (GUS) reporter construct using biolistic bombardment and priming in leaves from A. arguta AA07_03 plantlets [35]. The GUS activity was measured 48 hours after DNA bombardment. Error bars represent the standard errors of the means for three independent biological replicates with six technical replicates each (n = 18). HopI1c was used as the positive control and un-infiltrated leaf tissue (Unshot) as the negative control. Tukey’s HSD indicates treatment groups that are significantly different at α ≤ 0.1 with different letters. (PDF) [file ppat.1010542.s010.pdf]

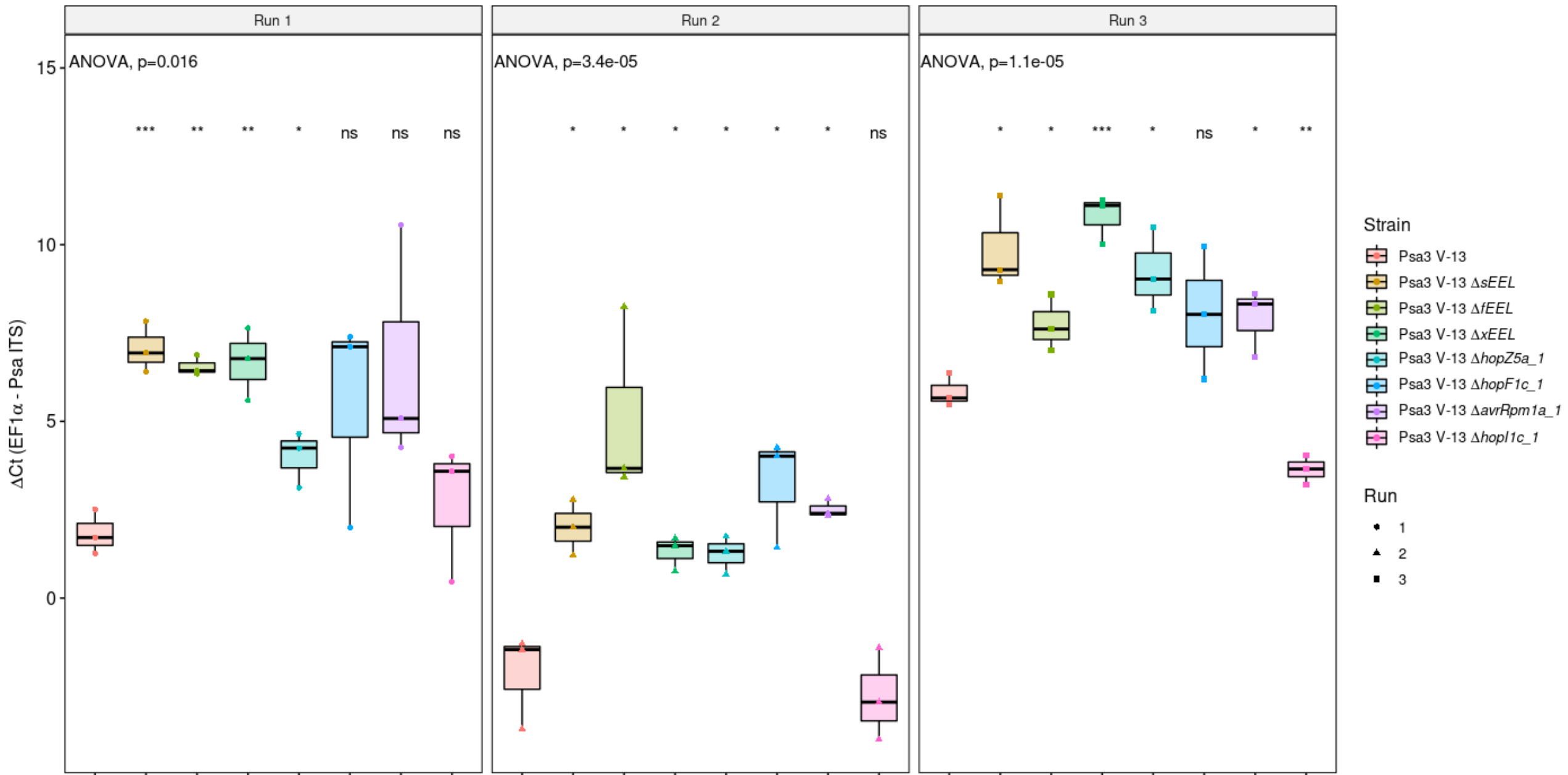

Supplement: S7 Fig — A. arguta AA07_03 kiwifruit plantlets were flood-inoculated at approximately 106 cfu/mL. Bacterial pathogenicity was quantified relative to Psa3 V-13 using the ΔCt analysis method for four pseudobiological replicates, per strain, per experimental run. Data are presented as box and whisker plots, with black bars representing the median values and whiskers representing the 1.5 inter-quartile range. The data have been faceted by experimental run. Asterisks indicate the statistically significant difference of Student’s t-test between the indicated strain and wild-type Psa3 V-13, where p ≤.05 (*), p≤.01 (**), p≤.001 (***), and p>.05 (ns; not significant). These three experiments (biological replications) were separately conducted with three batches of independently grown plants. (PDF) [file ppat.1010542.s011.pdf]

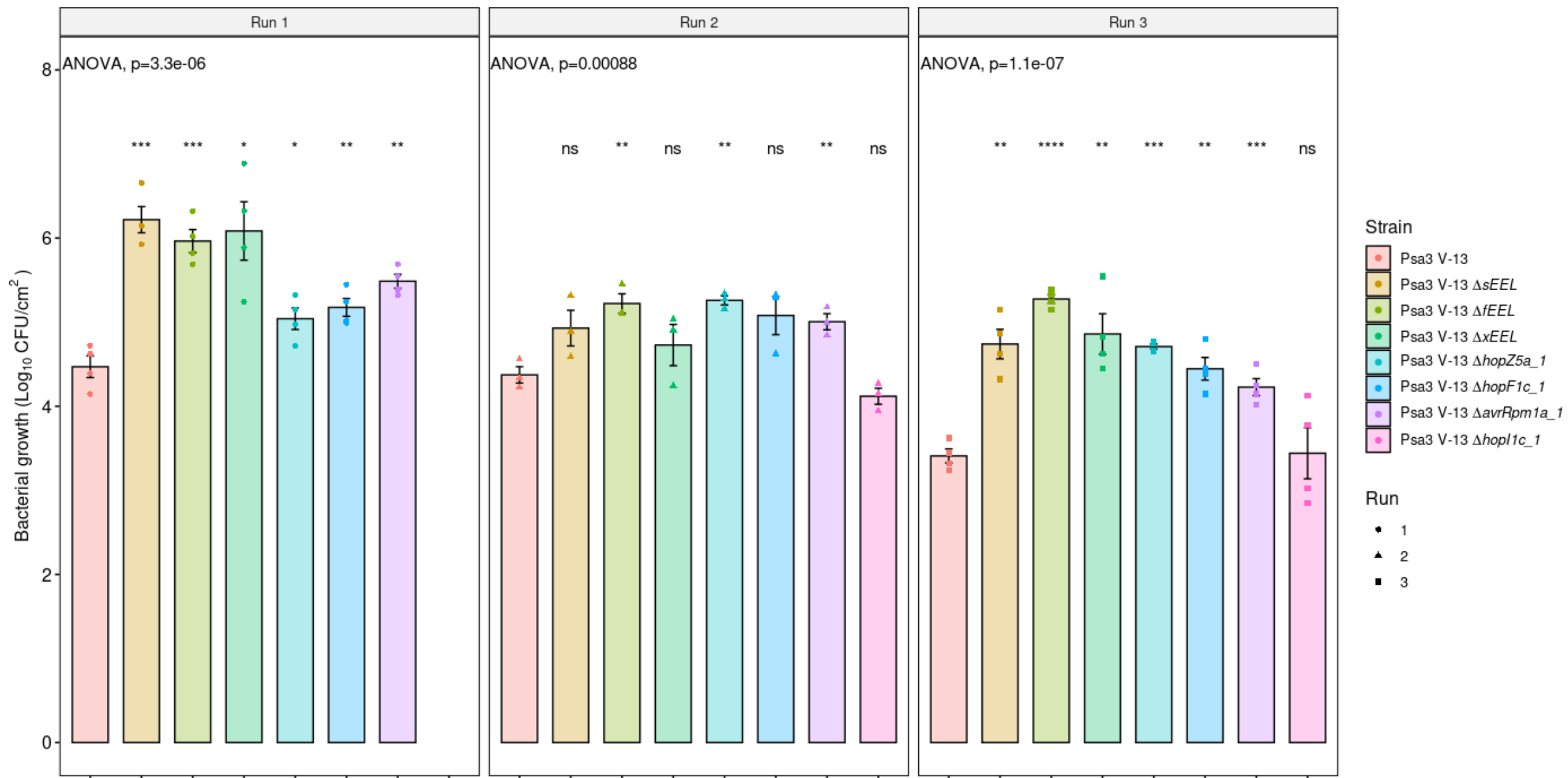

Supplement: S8 Fig — A. arguta AA07_03 kiwifruit plantlets were flood-inoculated at approximately 106 cfu/mL. Bacterial pathogenicity was quantified relative to Psa3 V-13 using plate count quantification for four pseudobiological replicates, per strain, per experimental run. The data have been faceted by experimental run. Asterisks indicate the statistically significant difference of Student’s t-test between the indicated strain and wild-type Psa3 V-13, where p ≤.05 (*), p≤.01 (**), p≤.001 (***), and p>.05 (ns; not significant). These three experiments (biological replications) were separately conducted with three batches of independently grown plants. (PDF) [file ppat.1010542.s012.pdf]

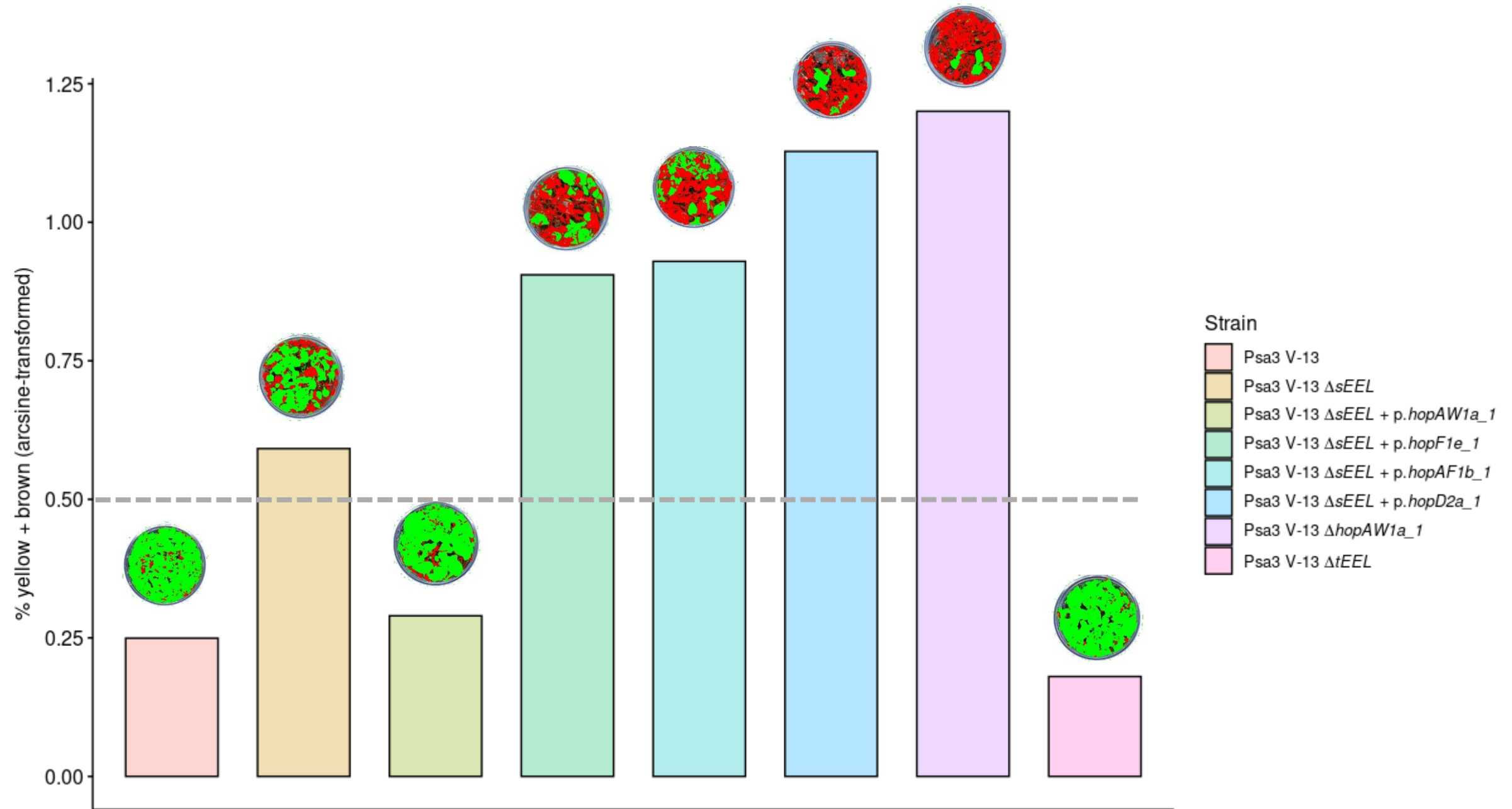

Supplement: S10 Fig — A modified PIDIQ image-based analysis of leaf yellowing and browning, expressed as a normalized arcsine-transformed percentage for symptomology photographs taken at 50 days post-infection (S9 Fig). Methodology adapted and modified from that of Laflamme, Dillon [70]. (PDF) [file ppat.1010542.s014.pdf]

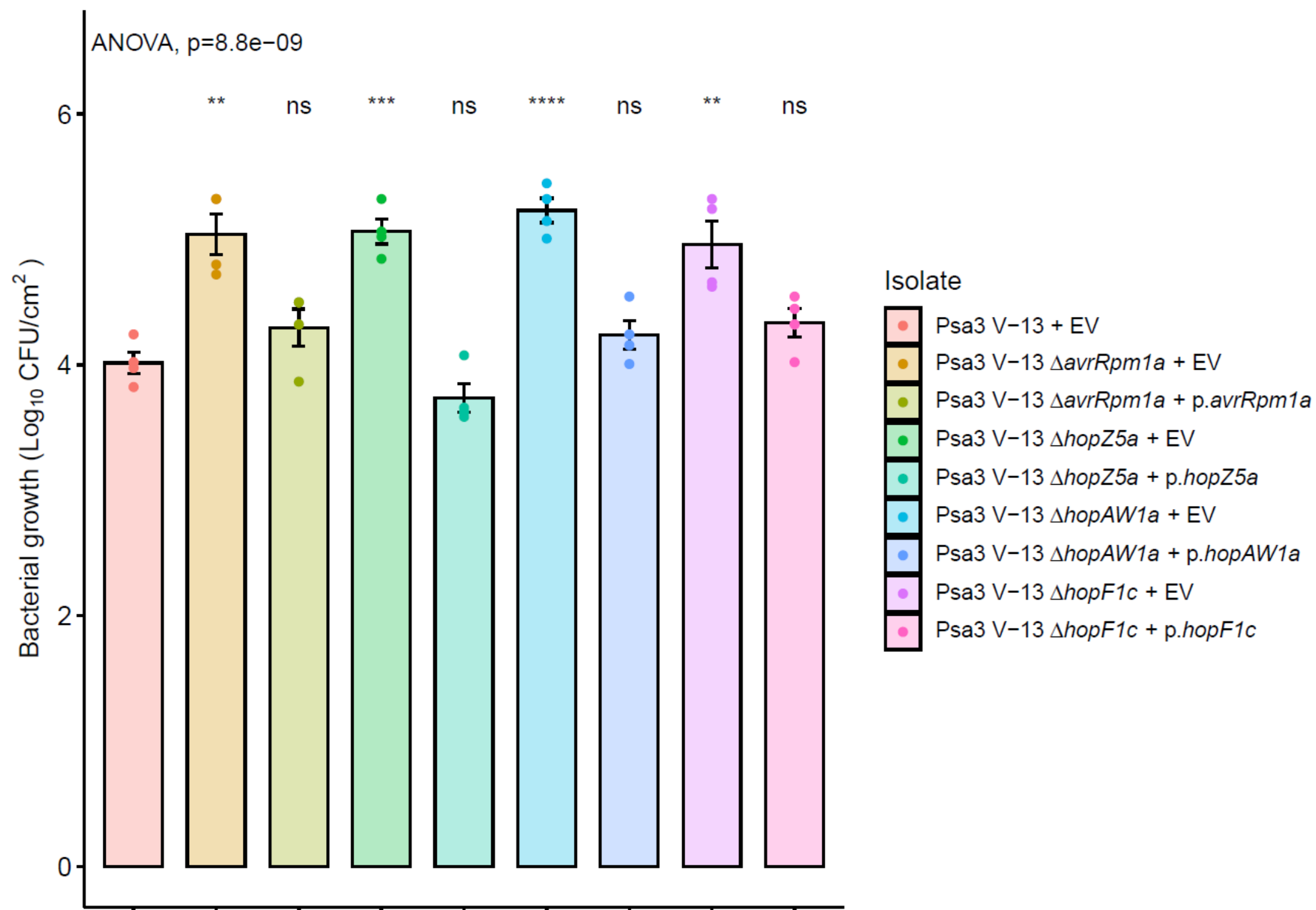

Supplement: S11 Fig — sEEL effectors in cloned binary vector constructs tagged with GFP, or an empty vector (Control), were co-expressed with a β-glucuronidase (GUS) reporter construct using biolistic bombardment and priming in leaves from A. arguta AA07_03 plantlets [35]. The GUS activity was measured 48 hours after DNA bombardment. Error bars represent the standard errors of the means for three independent biological replicates with six technical replicates each (n = 18). Un-infiltrated leaf tissue (Unshot) was used as a negative control. Tukey’s HSD indicates treatment groups which are significantly different at α ≤ 0.1 with different letters. (PDF) [file ppat.1010542.s015.pdf]

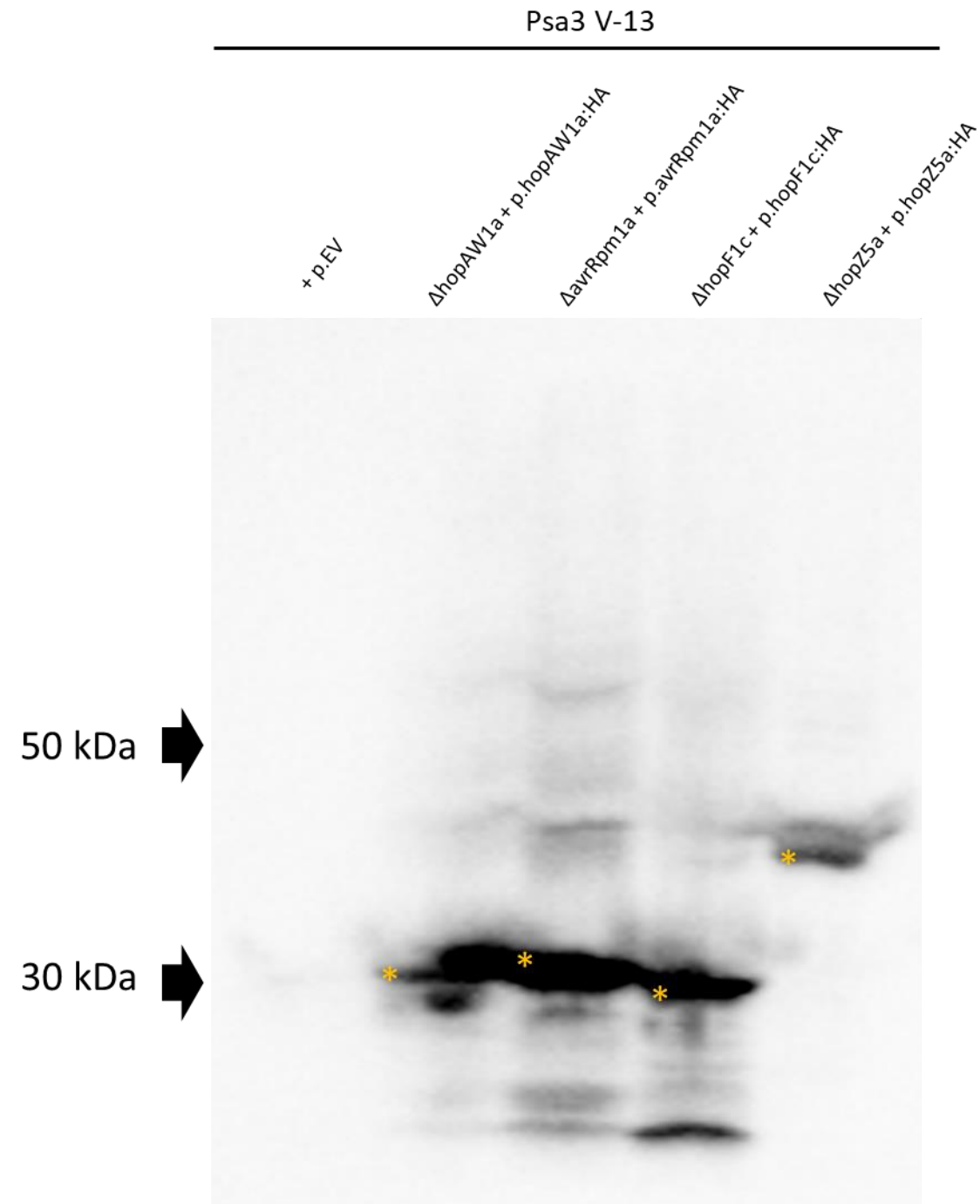

Supplement: S12 Fig — A. arguta AA07_03 kiwifruit plantlets were flood-inoculated at approximately 106 cfu/mL. Bacterial pathogenicity was quantified relative to Psa3 V-13 using plate count quantification. Bar height represents the mean log10 cfu/cm2 and error bars represent the standard error of the mean (SEM). (PDF) [file ppat.1010542.s016.pdf]

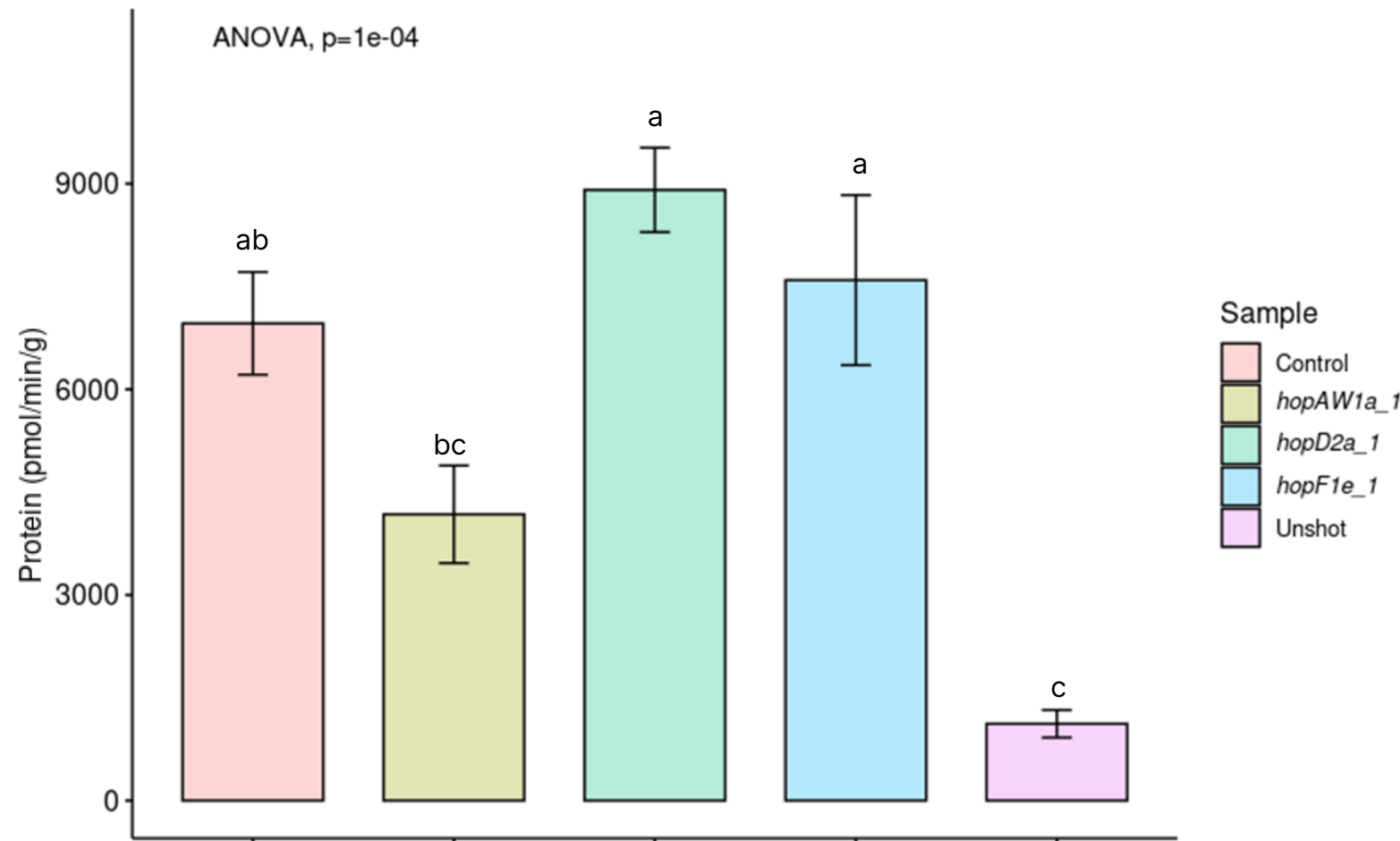

Supplement: S13 Fig — Wildtype Psa3 V-13 carrying empty vector (+ EV), or Psa3 V-13 avirulence effector knockout strains carrying the plasmid-borne type III secreted effector proteins tagged with 6 × HA (complemented strains) were diluted to 5 x 10^8^ cfu/mL in hrp-inducing liquid medium, cells pelleted at 6 hr post-inoculation by centrifugation at 12000 g, and supernatant boiled in 1x Laemmli buffer, and western blots conducted using α-HA antibody. Yellow asterisks indicate expected sizes for each tagged protein band. HopF1c is cloned and expressed with its preceding chaperone, ShcF. (PDF) [file ppat.1010542.s017.pdf]

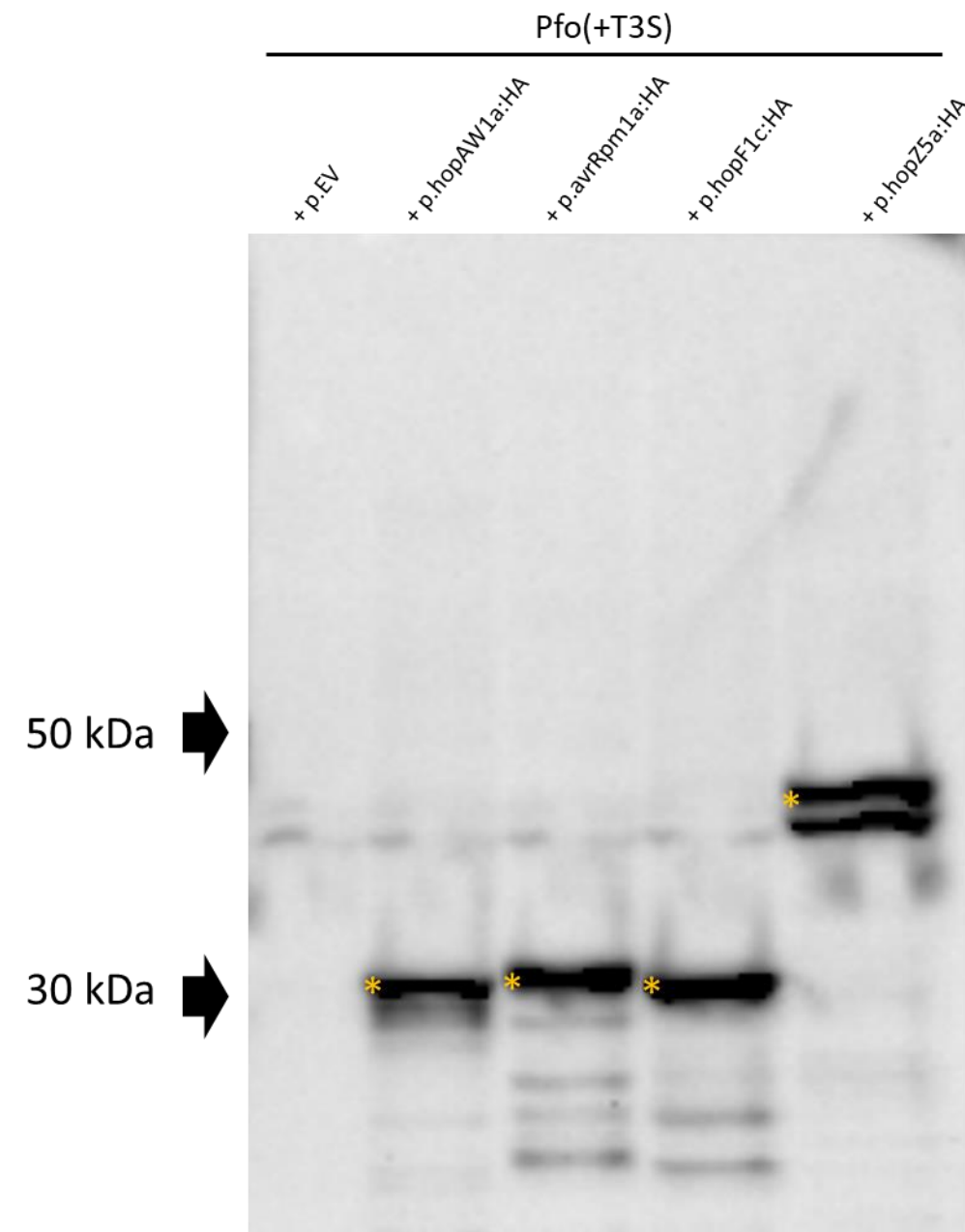

Supplement: S14 Fig — Pfo(+T3S) carrying empty vector (+ EV), or carrying the plasmid-borne type III secreted effectors from Psa3 V-13 tagged with 6 × HA were diluted to 5 x 10^8^ cfu/mL in hrp-inducing liquid medium, cells pelleted at 6 hr post-inoculation by centrifugation at 12000 g, and supernatant boiled in 1x Laemmli buffer, and western blots conducted using α-HA antibody. Yellow asterisks indicate expected sizes for each tagged protein band. HopF1c is cloned and expressed with its preceding chaperone, ShcF. (PDF) [file ppat.1010542.s018.pdf]

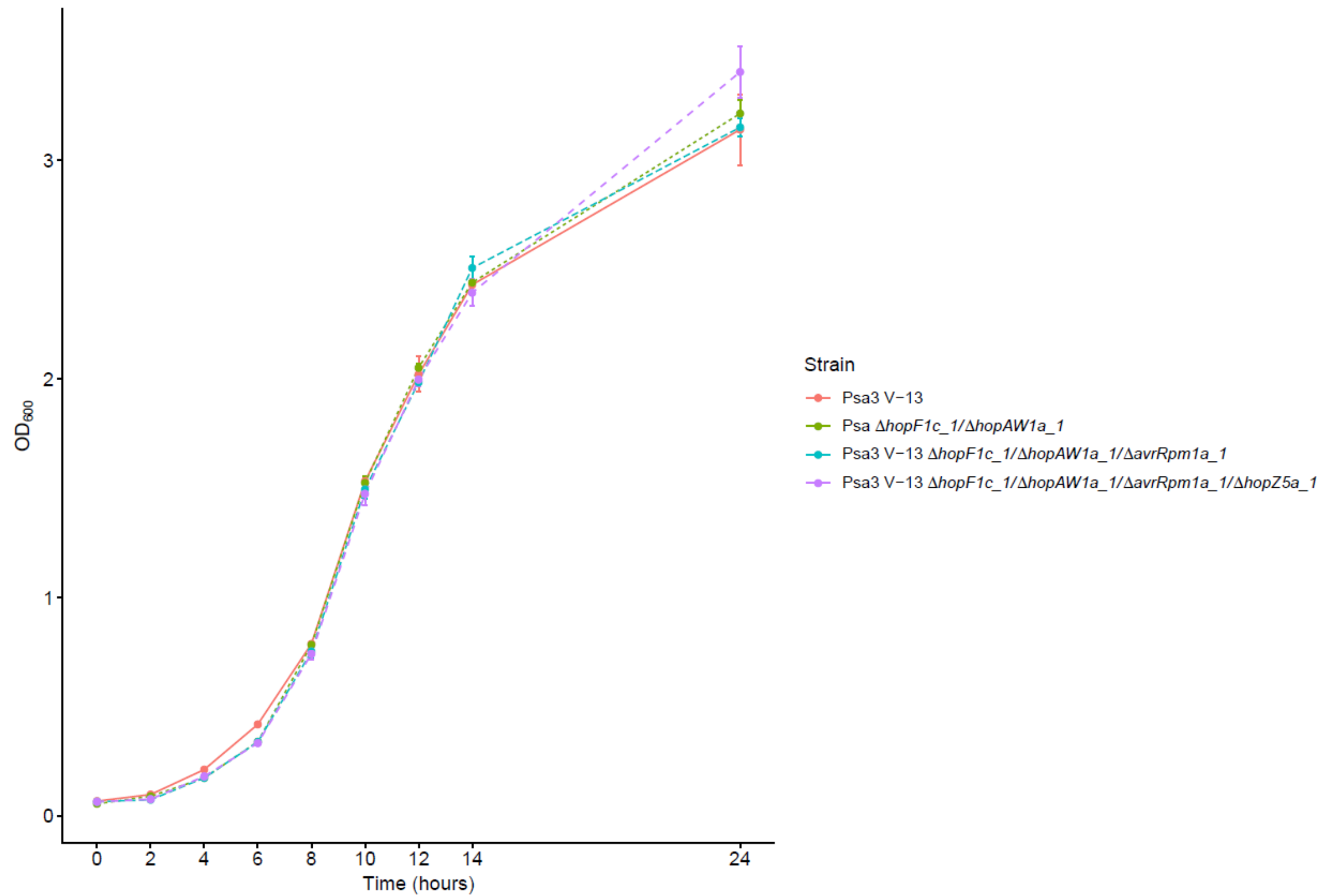

Supplement: S15 Fig — Points represents the mean OD600 and error bars represent the standard error of the mean (SEM) for three independent biological replicates. (PDF) [file ppat.1010542.s019.pdf]

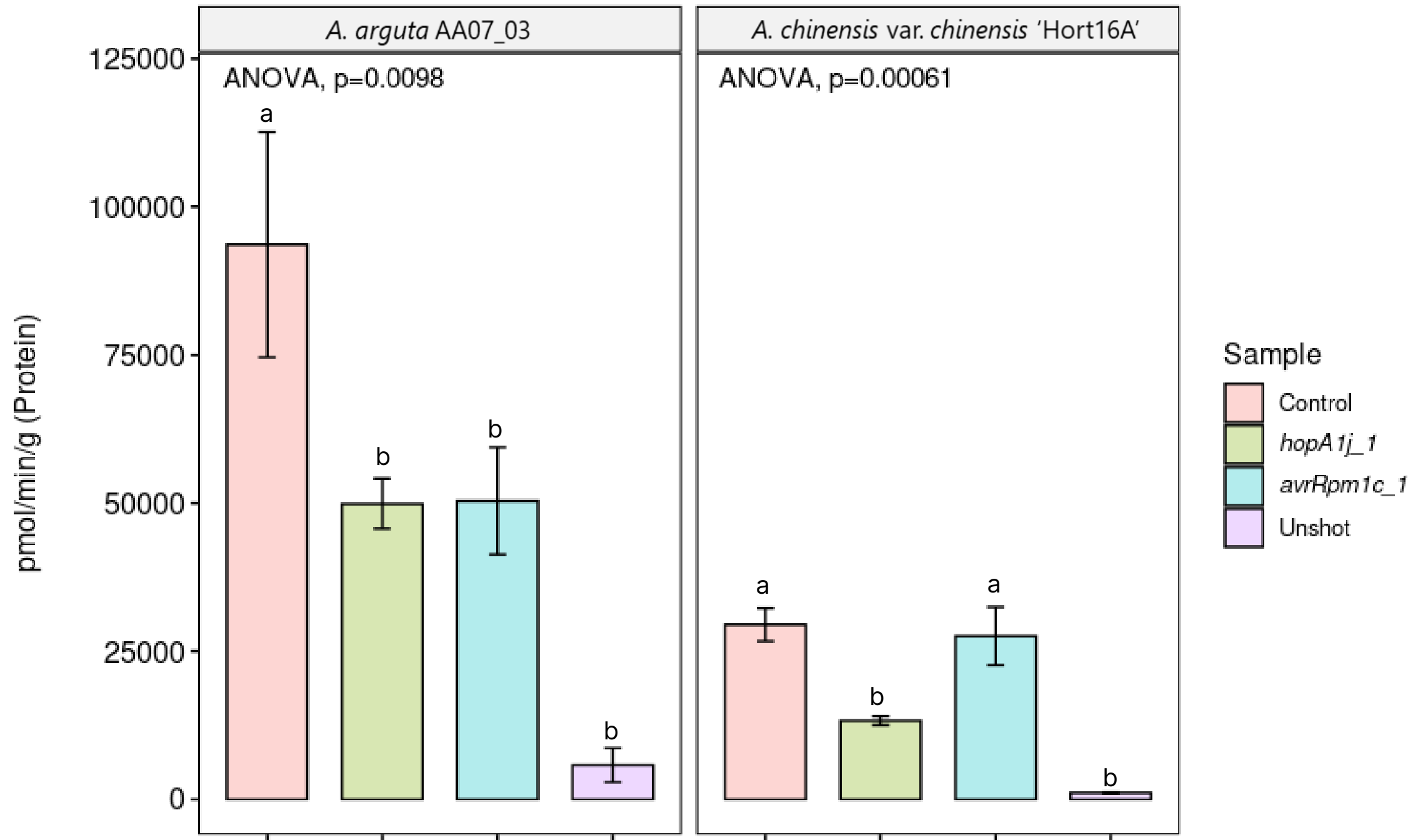

Supplement: S16 Fig — Effectors in cloned binary vector constructs tagged with Green Fluorescent Protein (GFP), or an empty vector (Control), were co-expressed with a β-glucuronidase (GUS) reporter construct using biolistic bombardment and priming in leaves from A. arguta AA07_03 plantlets [35]. The GUS activity was measured 48 hours after DNA bombardment. Error bars represent the standard errors of the means for three independent biological replicates with six technical replicates each (n = 18). HopA1 from Pseudomonas syringae pv. syringae 61 was used as the positive control and un-infiltrated leaf tissue (Unshot) as the negative control. Tukey’s HSD indicates treatment groups which are significantly different at α ≤ 0.1 with different letters. (PDF) [file ppat.1010542.s020.pdf]
