# Supplementary material for: Effector loss drives adaptation of Pseudomonas syringae pv. actinidiae biovar 3 to Actinidia arguta
Source: PLoS Pathog. 2022 May 27;18(5):e1010542. doi: 10.1371/journal.ppat.1010542 (PMC9182610; doi:10.1371/journal.ppat.1010542)

(Supplementary) Figure S9

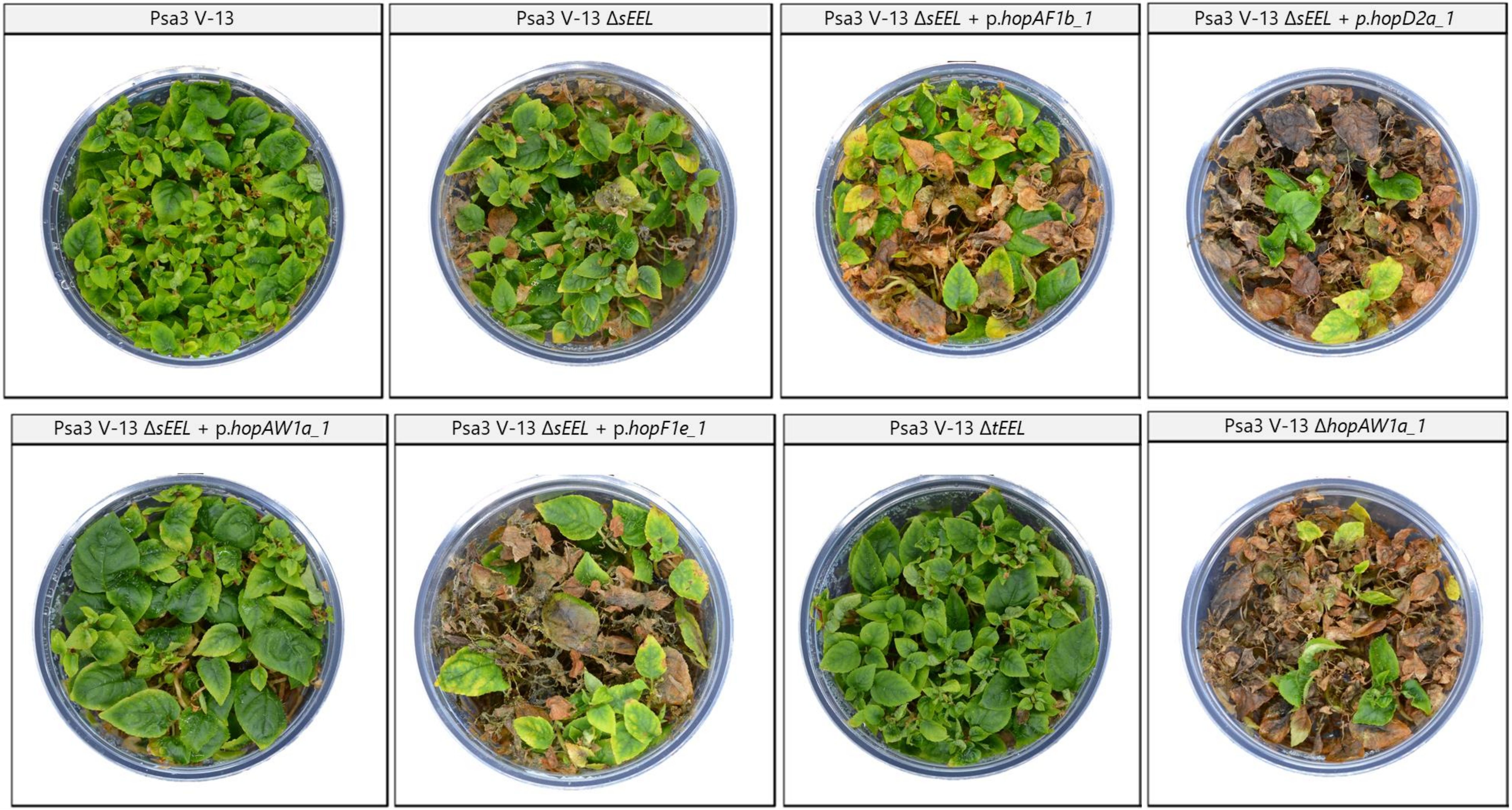

Supplement: S9 Fig — A. arguta AA07_03 kiwifruit plantlets were flood-inoculated at approximately 107 cfu/mL. Photographs of symptom development with representative pottles were taken at 50 days post-infection. (PDF) [file ppat.1010542.s013.pdf]
