# Supplementary material for: Effector loss drives adaptation of Pseudomonas syringae pv. actinidiae biovar 3 to Actinidia arguta
Source: PLoS Pathog. 2022 May 27;18(5):e1010542. doi: 10.1371/journal.ppat.1010542 (PMC9182610; doi:10.1371/journal.ppat.1010542)
Supplement: Attachment — Submitted filename: Response to reviewers_Arguta_PLoSpath.docx [file ppat.1010542.s021.docx]

The Editors

PLoS Pathogens

24-March-2022

**RE: manuscript ID: PPATHOGENS-D-21-02462 "** **Effector loss drives adaptation of *Pseudomonas syringae* pv. *actinidiae* biovar 3 to the non-host *Actinidia arguta*"**

Dear Prof. Coaker

We would like to thank the referees for their positive comments and very thorough reviewing of our manuscript. As requested, we have resubmitted a significantly-revised version that takes into account the editors’ and reviewers' requested suggestions. This has included performing a number of additional experiments which has required the addition of a co-author Minsoo Yoon. I have received Email confirmation from all authors approving this addition.

Below we provide a detailed list of our responses to the review comments and a description of the changes we have made in the manuscript. We have uploaded two versions of the revised manuscript: one with tracked changes denoting where the text has been changed (all line numbers described refer to this document); the other a clean version.

Reviewer's Responses to Questions

Our responses are highlighted in green.

General comments

More nuanced explanation for avr status of effectors and effectors working together. For example, loss of an effector causing reduced growth on a plant could be due to loss of the ability of an effector to suppress recognition of another. Include both ETI and failed ETS as a framework for compatibility.

We acknowledge the value of these comments and have discussed the possibility of ETI vs failed ETS in the following lines: 387-408; 492

Shorter the discussion and highlight major findings and implications more succinctly.

We have made significant changes to the discussion resulting in a reduction of this section by ~25%

**Part I - Summary**

Reviewer #1: In this manuscript, Hamara et al assessed the ability of several strains and effector mutants thereof to cause disease and grow in kiwi and kiwiberry cultivars. They also used a particle bombardment assay to determine the ability of several effectors to be recognized. The strength of this manuscript is in its description of how they might be involved in host restriction/recognition. Many mutants were made and this allowed the authors to infer the function of several effectors. The weakness is that although hosts are compared, we don’t learn about whether resistance is single or multigenic and we don’t get much new insight about these effectors other than they may be triggering resistance. Furthermore, sometimes alternative explanations of the data were not made. For example, the loss of an effector causing reduced growth on a plant could be due to loss of the ability of an effector to suppress recognition of another effector). The analysis of the data limited the ability of the reader to assess how the mutants behaved relative to each other (not just relative to a WT strain). A non-specialist may likely have difficulty following the results presented, owing to the way the work is presented and the lack of clear explanations of some of the assays. The discussion is long and often does not focus on the putting the major findings into context in a succinct way.

We have significantly re-written the Discussion to cover these insights as suggested by the Reviewer. We have also considerably reduced the discussion section to be more succinct and to cover the options of unmasked ETI and failed ETS in context of our findings. We feel that how these effectors trigger resistance is beyond the scope of this work.

Reviewer #2: The present manuscript submitted by Hemara et al describes a very interesting and useful study that certain T3Es in Psa3, including AvrRpm1a, HopF1c, HopZ5a and HopAW1a, might be recognized by Actinidia argute, but not by its relative A. chinensis, leading to species-specific resistance. The authors convincingly showed certain T3Es responsible for the resistance by screening of a complete library of Psa3 effector knockout strains, which could be further supported by the isolation of a natural EEL-mutant, which contains a 49 kb deletion and causes disease in A. argute. The experiments were well designed and performed with different setups from this group (Jayaraman et al., 2020; 2021), and it is easy to follow the context. Though the authors discussed in line 448-454, it seems A. argute is very resistant to Psa3, regarding the huge difference in bacterial numbers compared to A. chinensis, raising the question of contribution of ETI in A. argute as well as the resolution of the assays performed. The authors are suggested to address the questions below:

Reviewer #3: The authors have conducted a thorough series of genetic experiments to identify hopAW1a_1 as the genetic basis of Psa3 host rage expansion onto A. arguta based on a Psa3 natural genetic variant with a large deletion in its EEL region. In addition to hopAW1a_1, three other effectors are identified that contribute to decreased bacterial load in A. arguta. The group has invested extensively in the tools and techniques to work with this challenging host and the genetics are sound. With the exception of hopAW1a_1, I don't think I would have been so quick to assign avr status to the other effectors based on their quantitative contributions to load and compatibility but I imagine further study will resolve that. In addition, the authors propose lack of ETI as sufficient for compatibility when plainly that is insufficient. Using the zig zag model as a framework failed ETS is likely a factor here. The four combo mutant supports that interpretation.

Reviewer #4: The authors conducted a genome sequencing field survey and identified a strain of Psa3 (P. syringae pv. actinidiae biovar 3) that cause disease on the normally Psa3 resistant plant Actinidia arguta. This strain possesses a deletion of the exchangeable effector locus (EEL) which the authors demonstrate contributes to the avirulence of Psa3 on P. arguta, in particular HopAW1 from this locus. These are the first Psa3 avirulence genes to be identified in Actinidae. Further screening identified 3 additional effectors that contribute to avirulence. Interestingly, cumulative KOs of these effectors did not increase bacterial growth, likely due to their contribution to virulence. This would suggest a robust immune strategy that recognizes multiple Psa3 effectors that cannot readily be lost without compromising virulence. Overall this a very interesting study that has been well executed, but I do have some comments that should be addressed.

**Part II – Major Issues: Key Experiments Required for Acceptance**

Reviewer #1: (No Response)

Reviewer #2: (1) Psa3 does not grow too much in A. argute as revealed by low bacterial titer, compared to the same inoculation condition in A. chinensis var. chinensis. The difference of bacterial numbers between Psa3 WT and Psa3 △hrcC is low (Figure 6), while there is no Psa3 mutant that shows reduced virulence (Figure 3), even in Psa3 △CEL which was proved to be essential in bacterial pathogenicity in A. chinensis (Jayaraman et al., 2020) and in other pathosystem. This could be due to, not limited to, more robust PTI responses or other specialized secondary metabolites in A. argute. In Figure1, the authors are suggested to investigate transcriptional changes of some defense-related genes, to support specific activation of ETI. There is possibility that A. argute could restrict Psa3 growth with more robust PTI responses, please include Psa3 △hrcC as well.

We appreciate this comment, but in kiwifruit, we are not in the position of being able to distinguish between PTI and ETI as these assays are currently under investigation and development. It is also unclear that these two responses are completely distinguishable, especially in light of recent work highlighting interconnectedness (Kadota et al., 2019 New Phytol; Ngou et al., 2021 Nature; Yuan et al., 2021 Nature). The ability of *A. arguta* plants to be able to trigger an ETI-like defense response was explored previously including defence gene expression in response to both wildtype and *ΔhrcC* strains of Psa3 (Jayaraman et al., 2021).

(2) Figure 2F and 2G, the authors are suggested to include data from 0 dpi; from Figure 2G it seems the Psa3 X-27 and Psa3 V-13 △sEEL did not grow, while there is decrease of bacterial titer in Psa3 V-13 WT. Adding data from 0 dpi could help provide a clear picture.

Added as Supplementary Figure S1.

(3) For the assays using Pfo-delivered protein expression, the authors are suggested to provide protein accumulation data supporting that the missing observations of conductivity changes was not because of the missing protein accumulation (Figure 5).

Experiments provided and reported in Supplementary Figure S14.

Reviewer #3: No major issues noted

Reviewer #4: Figure 6B: The decrease in growth observed for the polymutants on the susceptible variety could be explained by a general decrease in bacterial fitness. The author’s should measure the growth of these mutants in vitro compared to the wild-type strain. Decreases in fitness could also explain why an additive increase in virulence was not observed when multiple Avr genes were knocked out.

Experiments performed in Supplementary Figure S15. There was no observed difference in bacterial fitness *in vitro* between the knockout isolates. We are confident that the differences in *in planta* growth reflect recognition by the host.

The authors' complemented the sEEL mutant with hopAW1a (Fig 3D) but did not complement the knock-outs of the other 3 identified avirulence genes (HopZ5a, HopF1c and AvrRpm1a; see Fig 3B). The authors' should complement these 3 mutants to show that resistance is restored. If effectors are epitope tagged, western blots should be conducted to confirm expression (including sEEL+hopAW1a).

Experiments performed as requested and reported in Supplementary Figures S12 and S13.

Figure 3: Should HopF2 be HopF1c? If so, why does the HopF1c behave differently in Fig 6B?

The effector was incorrectly named and has now been changed to HopF1c.The non-significance of the hopF1c knockout strain in Figure 6 is less clear to us. This second round of results (Figure 6) appear to have greater variation than when conducted earlier (Figures 3 & 4) masking within-run significance, but also resulting in less clear differentiation. We could exclude this sample since it is not required for the greater comparison of multi-knockout strains, but we chose to include it so the variation between experiments may be reflected.

**Part III – Minor Issues: Editorial and Data Presentation Modifications**

Reviewer #1: Figure 1 comments
It is difficult to assess this data because of the different timing used and the lack of quantification. I suggest showing the Psa3 infection for A. chinensis at 1 day and also show the cleared tissue. Presumably, this analysis was done in multiple leaves on different plants (it’s not specified how many), so the data can be quantified in some way- counting brown spots or patches of brown leaves/area or similar. The callose experiments needs a water control, although I’m not sure this measurement is that informative without quantification of some kind. Please add a size bar to part A of the figure.

The aim of these experiments was to visualise the hypersensitive response (HR) in *A. arguta*. We were not attempting to quantify the response in any way. HR was demonstrated quantitatively in kiwifruit in our previous publication (Jayaraman et al 2021). The images in Figure 1A were taken using a binocular microscope we have added the magnification in the figure legend.

Fig 2, How many days until plants were photographed? How many plants looked like the picture shown?
For panel C, there is a faint band for V13. This is never discussed in the main text – why was it used, do we expect to see a band? Or is this supposed to be V13delsEEL?

Photographs were taken at 6 months post-infection as described in the Methods section (under “Rooted plant inoculations and testing”). The displayed leaf is representative of every infected leaf from the three plants.

The band referred to is a weak non-specific amplification present in all samples. This comment has now been added to the figure legend.

p.8 line 165 The authors say…The bacteria were not as virulent in A. arguta AA09_3 as they were in “Hort16A’ – here the authors should refer to Fig S1. It might be better to say the disease was more severe on Hort16A, since the authors did not measure the bacterial growth, which I usually consider important for assessing virulence.

Reference to Supplementary Figure S2 has been added. We consistently refer to virulence as readout from plant symptomology and pathogenicity as a read out of host colonization (bacterial counts). This is consistent with other research in the field, including mammalian pathogen work.

For the general reader, it would be a good idea to explain the biolistic assay briefly in the text the first time that you do it. I assumed this was based on the GUS interference assay originally developed by the Katagiri lab. It won’t be intuitive to a non specialist that effectors that are recognized suppress GUS expression. Later in the ms the authors discuss using suppression of GUS activity, was this the same assay used in the early part of the paper (eclipse)? If so, the authors should explain it earlier in the paper. I think recognition should be distinguished from the hypersensitive response, which usually means cell death. Its not clear that GUS expression is prevented by cell death versus some other aspect of recognition. The authors mention their surprise at the lack of ion leakage (line 269, 270), but by now there are a number of examples in the literature where recognition occurs without cell death in a resistance gene-dependent manner.

This is based on a previous development paper that was not cited. We have included a brief description and cited the relevant works. (Lines 205-206)

Fig 6
Why is B plotted from Log10 of 0 to 12.5? It makes detecting differences on the graph very difficult. I recommend using no more than log10 of 10 for the y axis. I think that the authors should be cautious about whether the mutations are additive or not, since they did not do a time course of infection. It’s also possible that these effectors are recognized by the same mechanism, which would explain why they are not additive. The authors should do an analysis to compare all strains to each other if they want to say one mutant grows less or more than another. It looks from the graphs that all strains were compared to WT -to decide whether each one was different from WT. Looking at the graph in B for Hort16A, the authors may consider that reduced growth could be due to unmasking of an avirulence effector, which looks most obvious for the strain lacking HopZ5a. Considering that another member of the HopZ family can suppress multiple avirulence effectors, it’s possible that this is what is happening for HopZ5a.

The Y axis has been changed as requested. We have removed mention of comparison between strains. While it is tempting to do so, the statistical analyses (not included) do not support conclusions based on comparisons between the multiple-KO strains.

Line 358, suggest deleting “Important”

“Important” deleted

Reviewer #2: 1) Figure 2C and 2E, please add size references for the DNA ladders, label and indicate the PCR bands with sizes specifically. In Figure legend, it should be (F-H), not (E-H).

These changes have been made.

2) Line 165-167 and Figure S1, it seems Psa3 X-27 and Psa3 V-13 △sEEL cause severer disease symptom in A. chinensis var. chinensis ‘Hort16A’, compared to Psa3 V-13, which is more pronounced in Psa3 V-13 △sEEL (Figure S1 and Figure S2). Does this suggest that sEEL contributes to the recognition in A. chinensis var. chinensis ‘Hort16A’ as well.

The 50 day photographs of plants are not intended to quantitate pathogenicity. They are there purely to illustrate the extent of disease after a prolonged period of infection. The variation in symptomology between biological reps is consequently high. We are therefore reluctant to make the suggested conclusion.

3) Figure S4 legend, please add information for taking samples of how many days post-inoculation.

This data is now presented in Supplementary Figure S5. The sampling detail (at 12 dpi) has also been included.

Reviewer #3: Line 86: Could you clarify non-syntenic in this context? non-syntenic compared to what?

The text has been changed to indicate that the lack of synteny is in comparison to other *P. syringae* genomes. (Lines 93-94)

Line 122: Please include a citation for MITEs, mention the DDE repeats and include a citation.

We have included both in the description and added a reference to MITEs. There are currently no references for Psa regarding DDE elements for recombination. However, we have also included a reference for DDE (and other insertion elements) in general, and added the insertion sequence name and Pfam number for clarity.

Line 168: Yes ETI could be a factor but there's likely more to host compatibly than failure to elicit ETI.
ETS-failure is the alternative hypothesis.

We appreciate this important comment. However, our description reflects the rationale for the next set of experiments rather than a discussion point on the role of ETI. We have discussed more in lines: 387-408, 492

Line 214: While I appreciate the desire for precision, referencing the two Actinidia species only by their cultivar designations or by the complete species w pathovars designation makes things harder on the reader. Particularly since single cultivars of each are primarily used throughout and cultivar comparisons are not part of this work. I'd suggest either using only "A. arguta' after the first introduction or other practical abbreviations eg Achn Aarg

We prefer to refer to the specific line since there is considerable variation within the *A. arguta* species.

Line 223: Plasmid loss is one possible explanation but it doesn't seem likely based on the success of the 50-day symptom experiments. This could have been assessed using either plates counts based on the selectable marker or RT-qPCR rather than being speculated. This feels like handwaving rather than a legitimate explanation. It isn't a problem to report observations partially inconsistent with the simple model. Also, partial resistance implies an additive rather than a co-dependence relationship.

Our work currently describes, using the plate count method, that plasmid loss is indeed likely to be occurring. While we appreciate that the qPCR data is only partially inconsistent with the plate count data, we prefer to report this discrepancy. We feel that this serves as warning of the limitation of plasmid-based complementation studies using qPCR, since this methodology does not discriminate between strains that have lost the effector and ones that haven’t.

Line 333: Is non-host an appropriate term given the circumstances?

We have removed the reference to non-host here. We have however mentioned the possibility that non-host resistance exists as a complex phenotype later for discussion purposes. (Line 492)

Figure 2: The genetic nomenclature used in this manuscript is not intuitive and would be greatly aided by providing a more detailed diagram representing the full Psa3 V13 EEL region the X-27 deletion and the multiple EEL sub-deletions directly in the figure. This does not need to be to scale in the figure but should indicate the gene content. This needs to be in primary figures rather than in the supplemental and should be displayed graphically rather than being keyed in the legend.

Supplementary Figure S4 added

Fig S3: diamonds are white and grey

This has been corrected.

Fig 5: The pastel rainbow color scale system does not work as effectively in the scatter plot since the close colors make it difficult to distinguish. I'd suggest moving Pfo WT results to a supp figure to expand the useful color distinctions.

We have split the figure into more panels to make it easier for the reader to interpret.

Fig 7:It would be preferable to indicate presence absence in these specific strains rather than generalized Psa biovar distributions

Changes made as suggested.

Reviewer #4: The results of Fig 3B suggest that all four avirulence genes are required for the resistance observed in the Psa3 V-13 wild type strain since individual mutations result in increased growth. Although polymutants do not appear to show cumulative increases in bacterial growth, likely due to compromised virulence, the results of Fig 3B show that the ETI responses are additive since the 4 avirulence genes found in the wild-type strain provides more resistance than the 3 found in the individual mutants. This aspect of additivity should be discussed.

We assume the reviewer means figure 4B. We appreciate this significant point and have added it to the Discussion. (Lines 384-386)

The X-axis of bar graphs should be labeled throughout the manuscript, as done in Figure 3, since it is difficult to follow the colors provided in the Figure Legends (eg. Fig 4, etc…).

We prefer using a key rather than labelling the X-axis. This maximises plot area, and minimises space wasted by duplicating labels.

Title: Given that strains of this pathovar Psa3 can cause disease on A. arguta the authors should define what they mean by non-host.

This has been changed to remove “non-host” given the wider discussion.

The authors should provide background information about the Psa3 strain in the introduction since it is the main focus of the manuscript.

Paragraph added lines 53-58

Line 66: What is meant by the “super pan effector repertoire” and how is it different from the “pan effector repertoire”?

For our purposes, this distinction is not significant. Therefore, we have removed mention of the “super” pan effector repertoire.

Line 139: What is the rationale for the naming the EEL mutants? Does the ‘s’ in sEEL mutant mean anything? Also, the author’s clarify that Psa3 V-13 is the wild-type strain.

We have clarified the different EELs in Supplementary Figure S4.

Line 161: “Psa3 X-27 and Psa3 V-13 ΔsEEL both produced similar disease symptoms to Psa3 V-13 in ‘Hort16A’…”. It appears that Hort16 is more susceptible to the sEEL mutant (ie. more disease). Does this indicate that the sEEL is also recognized in this variety?

As commented above: The 50 day photographs of plants are not intended to quantitate pathogenicity. They are there purely to illustrate the extent of disease after a prolonged period of infection. The variation in symptomology between biological reps is consequently high. We are therefore reluctant to make the suggested conclusion.

Line 166: “Knocking out the sEEL locus increased virulence in A. arguta AA07_03 quantitatively, but Psa3 X-27 or Psa3 V-13 ΔsEEL were not as virulent in A. arguta AA07_03 as they were in ‘Hort16A’.”

This is confusing since Psa3 X-27 appears to be more virulent on AA07_03 than on Hort16A according to FigS2.

See previous comment

Comparing the growth of sEEL on these two varieties supports the authors’ hypothesis that “there may be additional effectors recognized by AA07_03 within the Psa3 V-13 effector complement.”

Line 187: Which strains does “These” refer to?

Corrected to read these mutants

Line 242: HopF1e reduces bacterial growth when expressed in the sEEL mutant (Fig 4D) but appears to enhance disease symptoms (Fig S8). Why this discrepancy?

It is unclear why HopF1e reduces growth when plasmid-complemented but does not affect symptomology. However, it is clear from subsequent work that HopF1e does not contribute significantly to avirulence conferred by effectors from the sEEL locus. The decrease in counts for the plasmid-complemented strain may be an artefact, or can be explained if other effectors from the sEEL (HopD2a, HopAF1f, or HopF1a are able to suppress/interfere with recognition of HopF1e).

We trust we have answered the questions from the referees fully and to your satisfaction.

Sincerely

Matt Templeton
